# Determination of Equine Behaviour in Subjectively Non-Lame Ridden Sports Horses and Comparison with Lame Sports Horses Evaluated at Competitions

**DOI:** 10.3390/ani14121831

**Published:** 2024-06-20

**Authors:** Sue Dyson, Danica Pollard

**Affiliations:** 1 Independent Researcher, Church Road, Market Weston, Diss IP22 2NX, Suffolk, UK; 2 Independent Researcher, Rodham Road, Christchurch, Wisbech PE14 9NU, Cambridgeshire, UK; drdee.pollard@gmail.com

**Keywords:** Ridden Horse Pain Ethogram, lameness, canter, conflict behaviour, poor performance

## Abstract

**Simple Summary:**

The Ridden Horse Pain Ethogram (RHpE) comprising 24 behaviours was developed to facilitate the identification of musculoskeletal pain. A RHpE score ≥8/24 indicates the likely presence of musculoskeletal pain. The RHpE was applied to 1358 horses in competition, with a comparison of behaviours in lame and non-lame horses. In a large majority of the non-lame horses, the head was still, the front of the head was in a vertical position or behind the vertical, the eyes were open with no exposure of the sclera, all of the tongue remained within the oral cavity, the bit was symmetrically positioned, and the tail was held straight and carried freely. Horses maintained a regular rhythm and speed in all paces, moving straight on two tracks. Non-lame horses initiated canter with the correct leading forelimb and did not change legs in front or behind; there were no spontaneous changes of gait, no repeated forelimb or hindlimb stumbling, and an absence of bilateral hindlimb toe drag. Horses followed the direction of the rider’s cues, with no spooking, and went forward willingly, with an absence of bucking or rearing. Recognition of how a non-lame horse usually behaves may enhance equine welfare and improve training practices.

**Abstract:**

The Ridden Horse Pain Ethogram (RHpE) was developed to facilitate the identification of musculoskeletal pain. The aim of the current study was to collate behavioural data using the RHpE from horses at competitions assumed by their owners and/or riders to be fit for competition. The objectives were to quantify the frequency of occurrence of behaviours in pain-free horses and those with lameness or abnormalities of canter and to determine any differences between disciplines and levels of competition. The RHpE was applied to 1358 horses competing in Grand Prix (GP) dressage (n = 211), 5* three-day events (TDE) (n = 137), or low-level one-day events (ODE) (n = 1010). The median RHpE score for all horses was 4 (interquartile range [IQR] 2, 5; range 0, 12) and the median lameness grade was 0 (IQR 0, 1; range 0, 4). The Kruskal–Wallis test, followed by Dunn’s test for pairwise comparisons, found a difference in median RHpE scores between low-level ODE and GP dressage (*p* = 0.001), but not between 5* TDE and low-level ODE (*p* = 0.09) or between GP dressage and 5* TDE (*p* = 1.00). The median RHpE score was highest for low-level ODE. The Chi-square/Fisher’s exact test identified a significant difference in prevalence of most of the 24 behaviours of the RHpE in non-lame compared with lame horses. Recognition of the behaviours of non-lame horses may improve equine welfare and performance, and rider comfort, confidence, and safety.

## 1. Introduction

It is important that all stakeholders in the equine industry are able to recognise how a normal horse should move and behave, and how pain can modify gait and behaviour, in order to safeguard equine welfare. It is clear from studies performed in Denmark [1], Sweden [2], Switzerland [3,4], and the United Kingdom [5,6] that riders and trainers are poor at recognising lameness or pain-induced abnormalities of canter. Previous work has focused on the assessment of ridden horses’ behaviour to facilitate recognition of musculoskeletal pain [7,8,9,10,11,12,13]. A Ridden Horse Pain Ethogram (RHpE) was developed comprising 24 behaviours; the majority of the behaviours of the RHpE were greater than ten times more likely to be seen in a lame horse versus a non-lame horse [7]. A total RHpE score of ≥8/24 is likely to reflect the presence of musculoskeletal pain, although some lame horses have a score of <8/24.

The development of objective methods of gait analysis and their widespread use in equine lameness assessments and analysis of gaits at pre-purchase examinations have begun to raise questions about what constitutes normality [14,15,16,17,18,19]. Measurable gait asymmetries outside the so-called normal ranges [20,21] have been identified in both Warmblood [18] and Standardbred [14,18] foals and in mature riding horses assumed by the riders to be working satisfactorily at both elite and non-elite levels [15,16,17,19]. It is not known with certainty whether this reflects innate non-pain induced asymmetry (sometimes referred to as motor laterality) [20,22], which may or may not predispose to subsequent development of pain-related gait changes, or is pain related. One way of differentiation may be through assessment of ridden horse behaviour, although tack fit for horse and rider and rider force distribution may be confounding factors.

The high frequency of occurrence of lameness in sports horses [1,2,3,4,5,6] and riding school horses [23,24] means that many riders may have been over-exposed to abnormal behaviours from their formative riding years, potentially normalising these features. Riders are poor at correctly interpreting equine behaviour [25,26,27]. The horse riding public need to be more aware of the features of a pain-free horse during ridden exercise to facilitate differentiation between non-lame horses and lame horses or those with pain induced by ill-fitting tack or a rider’s weight distribution.

The aim of the current study was to collate behavioural data using the RHpE from horses at competitions, assumed by their owners and/or riders to be fit for competition. All horses competing at international level Grand Prix dressage and at 5* three-day events had also undergone a mandatory in-hand horse inspection by the Ground Jury and the Veterinary Delegate and had been deemed fit to compete. Horses competing at national (sub-elite) Grand Prix dressage or lower-level one-day events had not undergone any independent inspection. The objectives were to quantify the frequency of occurrence of behaviours in pain-free horses and those with lameness or abnormalities of canter and to determine any differences between disciplines and levels of competition.

## 2. Materials and Methods

### 2.1. Data Collection

This study employs data that had been previously collected and analysed to determine the relationship between RHpE scores and competitive performance [28,29,30,31]. The studies had been approved by the Ethics Review Panel of the Royal College of Veterinary Surgeons (2020–26).

The data were collected prospectively either from video recordings acquired in a standardised way during Grand Prix dressage tests [29,30], during live assessment of horses warming up for the dressage phase of 5* three-day events [28], or during the dressage tests of horses competing at lower-level (British Eventing 90, 100, and Novice) one-day events [31]. A good agreement of application of the RHpE between live horse assessment and video assessment of horses, performed by a trained observer, has previously been demonstrated [32].

The RHpE was applied and the results were recorded on individual purpose-designed forms before transfer to a Microsoft Excel (Office 365, Version 2310; Microsoft Corporation, Redmond, Washington, DC, USA) spread sheet. The presence of forelimb or hindlimb lameness and whether it was continuous or episodic, and if episodic under what circumstance it was observed, were recorded. Lameness was assigned a grade of 0 to 8 (0 = non-lame, 1 = very mild (just perceptible), 2 = mild, 3 = mild–moderate, 4 = moderate, 5 = moderately severe, 6 = severe (although the limb is loaded), 7 = partial weightbearing and 8 = non-weightbearing) [33]. A horse with a consistent lack of hindlimb impulsion and engagement was categorised as lame, but if bilaterally symmetrical, was not assigned a lameness grade. The presence of abnormalities of canter characterised by a stiff, stilted gait was noted; being on the forehand, lacking a suspension phase (a so-called four beat canter), having close temporal and spatial separation of the hindlimbs, or repeatedly changing legs behind were also recorded [34]. Noseband and bit types and the use of spurs were also documented.

### 2.2. Data Analysis

All statistical analyses were conducted using R Statistical Software (v4.3.1; R Core Team 2023) [35]. Ordinal variables (RHpE score and lameness grade) were described as medians with interquartile range (IQR) and range. Categorical variables were described as proportions (%). Data were described overall, by event type (Grand Prix Dressage, dressage phase of 5* three-day events [TDE] and dressage phase of low-level one-day events [ODE]) and by lameness categories.

#### 2.2.1. Event Type and Lameness

The Chi-square test of independence (or Fisher’s exact test if any count in the contingency table was ≤5) was used to assess the relationship between event type and presence of lameness (non-lame, lame, or abnormal canter), lameness frequency (non-lame, continuous lameness, or episodic lameness), and lameness presentation (non-lame, forelimb lameness, hindlimb lameness, or both forelimb and hindlimb lameness). Additionally, Cramér’s V was calculated to estimate the effect size (ES) measure for the Chi-square test. The strength of association for a Chi-square test with ≥4 degrees of freedom (df) was interpreted as suggested by Cohen [36]: weak if ES < 0.15, moderate if 0.15 < ES < 0.25 and strong if ES ≥ 0.25. The Kruskal–Wallis rank sum test, followed by Dunn’s test to assess pairwise comparisons (with *p*-values adjusted using Bonferroni adjustment), were used to assess the relationship between event type and median lameness grade and RHpE score. The eta-squared measure was calculated to estimate the effect size based on the H-statistic [37]. The ES was interpreted as: <0.06 as weak, 0.06 < ES < 0.14 as moderate and ≥0.14 as strong [38]. Results were considered significant where *p* < 0.05.

#### 2.2.2. Frequency of Occurrence of Each RHpE Behaviour and Event Type

The Chi-square/Fisher’s exact test was used to assess the relationship between the presence of each of the 24 behaviours of the RHpE and event type. Cramér’s V was calculated to estimate the ES measure for the Chi-square test. The strength of association for a Chi-square test with df = 2 was interpreted as suggested by Cohen [36]: weak if ES < 0.21, moderate if 0.21 < ES < 0.35 and strong if ES ≥ 0.35. Additionally, a Bonferroni adjustment was applied to the Chi-square *p*-values to adjust for multiple comparisons and results were considered significant where *p* < 0.002 (0.05/24).

#### 2.2.3. Frequency of Occurrence of Each RHpE Behaviour and Presence of Lameness or Abnormal Canter

The Chi-square/Fisher’s exact test was used to assess the relationship between the presence of each of the 24 behaviours of the RHpE and the presence of lameness, including a Bonferroni adjustment for multiple comparisons; results were considered significant where *p* < 0.002 (0.05/24). Cramér’s V was calculated to estimate the ES measure for the Chi-square test as above for a Chi-square test with df = 2.

#### 2.2.4. Frequency of Occurrence of the 24 Behaviours of the Ridden Horse Checklist for Sports Horses without Musculoskeletal Pain

For those horses with no detectable lameness, the frequency of occurrence of each of the 24 behaviours of the RHpE was calculated and the results used to determine the frequency of reverse features (for example, no rearing), in order to create a Ridden Horse Checklist for sports horses without musculoskeletal pain.

## 3. Results

Data were available for a total of 1358 horses. Of these, 15.5% (n = 211) participated in Grand Prix dressage tests, 10.1% (n = 137) participated in the dressage phase of 5* TDE, and 74.4% (n = 1010) participated in the dressage phase of low-level ODE.

### 3.1. Descriptives of All Horses

Fifty-nine percent (n = 805) of horses showed no evidence of lameness. Forty-one percent (n = 553) of horses were identified as being lame, 18.8% (n = 255) were non-lame but had an abnormal canter, and 40.5% (n = 550) were non-lame and had a normal canter. Of the lame horses, 87.0% (n = 481) exhibited continuous lameness and 13.0% (n = 72) exhibited episodic lameness. The majority of the lame horses presented with hindlimb lameness (79.9%; n = 442), with 10.8% (n = 60) having forelimb lameness and 9.2% (n = 51) having both forelimb and hindlimb lameness.

The median RHpE score for all horses was 4 (IQR 2, 5; range 0, 12) and the median lameness grade was 0 (IQR 0, 1; range 0, 4). The three most commonly observed behaviours of the RHpE for all horses were: head behind vertical (>10°) for ten or more seconds (61.3%, n = 833); an intense stare for five or more seconds (44.9%, n = 610); and head being tilted repeatedly (38.4%, n = 521). The three least commonly observed behaviours of the RHpE for all horses were: gait being too slow (frequency of trot steps less than 35 in 15s) (0.0%, n = 0); rearing (both forelimbs off the ground) (0.9%, n = 12); and the eye lids closed or half closed for two to five seconds (1.0%, n = 14).

### 3.2. Event Type and Lameness or Abnormality of Canter

**Figure 1:**
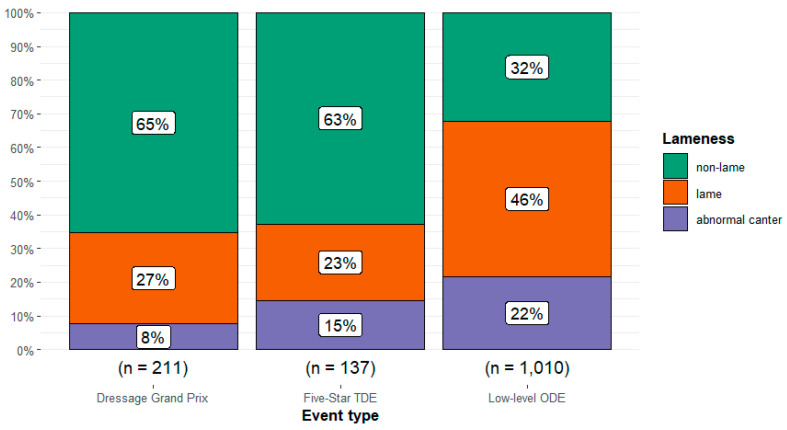
Observations of the proportion of all horses (n = 1358) without lameness and with a normal canter, with lameness, and without lameness but with an abnormal canter participating at three different event types: Grand Prix dressage, 5* three-day events (TDE), and low-level one-day events (ODE). In Grand Prix dressage and ODEs, horses were assessed during the dressage test; at TDEs, the horses were evaluated during warm-up for dressage.

There was a relationship between event type and presence of lameness (Χ^2^ =113.6, df = 4, *p* < 0.001) with a moderate effect size (Cramér’s V = 0.20 [95%CI 0.16, 0.24]) (Figure 1). The presence of lameness was highest in the low-level ODE event type (46.0%, n = 465/1010) and lowest in the 5* TDE event type (22.6%, n = 31/137). The presence of abnormal canter was highest in the low-level ODE event type (21.7%, n = 219/1010) and lowest in the Grand Prix Dressage event type (7.6%, n = 16/211).

**Figure 2:**
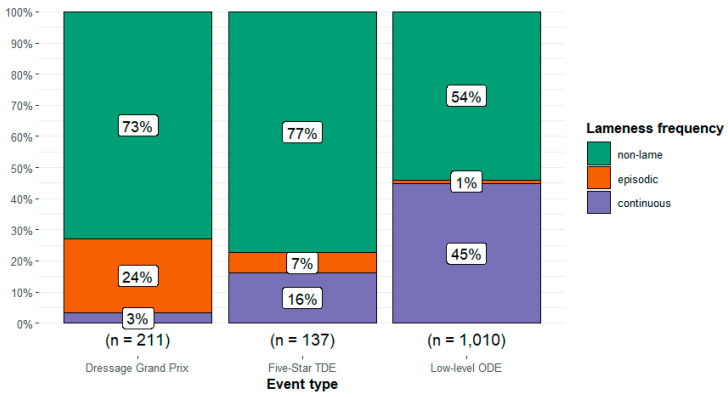
Observations of the proportion of all horses (n = 1358) without lameness, with episodic lameness, and with continuous lameness participating at three different event types: Grand Prix dressage, 5* three-day events (TDE), and low-level one-day events (ODE). In Grand Prix dressage and ODEs, horses were assessed during the dressage test; at TDEs, the horses were evaluated during warm-up for dressage.

There was a relationship between event type and frequency of lameness type (*Χ*^2^ = 285.6, df = 4, *p* < 0.001), with a strong effect size (Cramér’s V = 0.32 [95%CI 0.28, 0.36]) (Figure 2). The frequency of continuous lameness was highest in the low-level ODE event type (44.8%, n = 452/1010) and lowest in the Grand Prix Dressage event type (3.3%, n = 7/211). The frequency of episodic lameness was highest in the Grand Prix Dressage event type (23.7%, n = 50/211) and lowest in the low-level ODE event type (1.3%, n = 13/1010).

**Figure 3:**
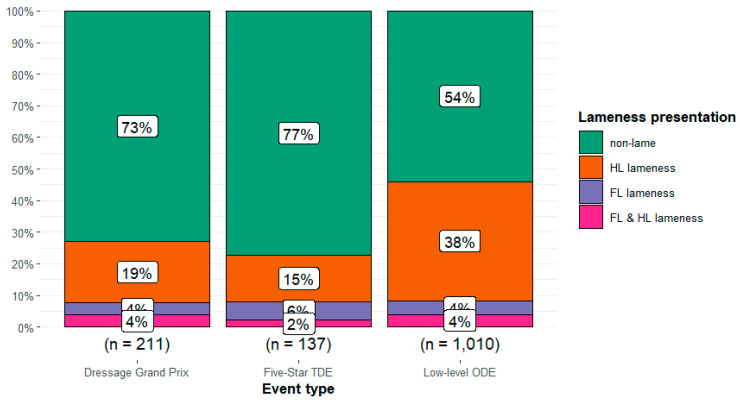
Observations of the proportion of all horses (n = 1358) without lameness, and with lameness presentation, in different sets of limbs (hindlimb, HL; forelimb, FL; FL and HL) participating at three different event types: Grand Prix dressage, 5* three-day events (TDE), and low-level one-day events (ODE). In Grand Prix dressage and ODEs, horses were assessed during the dressage test; at TDEs, the horses were evaluated during warm-up for dressage.

There was a relationship between event type and lameness presentation (*Χ*^2^ = 53.9, df = 6, *p* < 0.001) with weak to moderate effect size (Cramér’s V = 0.13 [95%CI 0.08, 0.17]) (Figure 3). The most common presentation of lameness, hindlimb lameness, was highest in the low-level ODE event type (37.7%, n = 381/1010) and lowest in the 5* TDE event type (14.6%, n = 20/137).

There was an association between lameness grade and event type (*p* < 0.001, ES = 0.02 [weak]) and RHpE score and event type (*p* < 0.001, ES = 0.01 [weak]) (Table 1). Pairwise comparisons revealed a significant difference in median lameness scores between low-level ODE and Grand Prix dressage (*p* = 0.002) and between 5* TDE and low-level ODE (*p* < 0.001), but not between Grand Prix dressage and 5* TDE (*p* = 0.59). Median lameness scores were higher for low-level ODE. Pairwise comparisons revealed a significant difference in median RHpE scores between low-level ODE and Grand Prix dressage (*p* = 0.001), but not between 5* TDE and low-level ODE (*p* = 0.09) or between Grand Prix dressage and 5* TDE (*p* = 1.00). The median RHpE score was highest for low-level ODE.

### 3.3. Frequency of Occurrence of Each RHpE Behaviour and Event Type

The frequency of occurrence for each of the 24 behaviours of the RHpE according to event type are summarised in Table 2. There was a moderate to strong association between mouth opening with separation of the teeth for ≥10s and event type (*p* < 0.001, Cramér’s V = 0.32 [95%CI 0.27, 0.38]). Mouth opening was observed most frequently in Grand Prix dressage. There was a moderate relationship between both moving on three tracks (*p* < 0.001, Cramér’s V = 0.27 [95%CI 0.22, 0.32]) and repeated stumbling or bilateral hindlimb toe drag (*p* < 0.001, Cramér’s V = 0.28 [95%CI 0.23, 0.34]) and event type. Both occurred most frequently in low-level ODEs. There was a weak to moderate association between repeated tail swishing and event type (*p* < 0.001, Cramér’s V = 0.17 [95%CI 0.12, 0.23]). Tail swishing was seen most frequently in Grand Prix dressage. There was a weak to moderate association between both repeated head tilt (*p* < 0.001, Cramér’s V = 0.19 [95%CI 0.13, 0.24]) and repeated exposure of the sclera (*p* < 0.001, Cramér’s V = 0.18 [95%CI 0.12, 0.23]) and event type. Both were most frequent at 5* TDE. There was a weak to moderate relationship between both the bit being pulled through to one side (*p* < 0.001, Cramér’s V = 0.19 [95%CI 0.13, 0.24]), and head movement up and down not being in synchrony with the trot rhythm (*p* < 0.001, Cramér’s V = 0.18 [95%CI 0.12, 0.23]), and event type. Both were predominantly observed at low-level ODE.

### 3.4. Frequency of Occurrence of Each RHpE Behaviour and Presence of Lameness or Abnormal Canter

The frequency of occurrence of each of the behaviours of the RHpE according to lameness status are summarised in Table 3. There was strong association between the occurrence of repeated stumbling or bilateral hindlimb toe drag and gait abnormalities (*p* < 0.001, Cramér’s V = 0.49 [95%CI 0.44, 0.54). There was a moderate association between ears back for ≥ 5s and gait abnormalities (*p* < 0.001, Cramér’s V = 0.29 [95%CI 0.23, 0.34]). There was a moderate to strong association between an intense stare for ≥ 5s and abnormalities of gait (*p* < 0.001, Cramér’s V = 0.30 [95%CI 0.25, 0.36]). There was a weak to moderate relationship between the bit being pulled through to one side (*p* < 0.001, Cramér’s V = 0.16 [95%CI 0.10, 0.21]), and moving on three tracks (*p* < 0.001, Cramér’s V = 0.20 [95%CI 0.15, 0.26]) and gait abnormalities. Each of these features was most prevalent in lame horses.

### 3.5. Prevalence of the 24 Behaviours of the Ridden Horse Checklist for Sports Horses without Musculoskeletal Pain

The prevalence of the “reverse” behaviours of the RHpE, the Ridden Horse Checklist for horses without lameness, is summarised in Table 4. It is notable that only 38.9% of non-lame horses did not have the head behind vertical >10° for ≥ 10s, and only 66.0% had the mouth shut with the teeth apposed.

## 4. Discussion

### 4.1. The Ridden Behaviour of Non-Lame Horses

This study of competition horses demonstrated that in a large majority of the non-lame horses the head was still (did not move up and down or from side to side), the front of the head was in a vertical position or behind the vertical, the eyes were open with no exposure of the sclera, all of the tongue remained within the oral cavity, the bit was symmetrically positioned and the tail was held straight and carried freely. Horses maintained a regular rhythm and speed in all paces, moving straight on two tracks. Non-lame horses initiated canter with the correct leading forelimb and did not change legs in front or behind; there were no spontaneous changes of gait, no repeated forelimb or hindlimb stumbling, and an absence of bilateral hindlimb toe drag. Horses followed the direction of the rider’s cues, with no spooking, and went forward willingly, with an absence of bucking or rearing.

### 4.2. Gait Abnormalities in Competition Horses

Overall, in this population of competing horses the median lameness grade was 0/8, with the highest prevalence of lameness (46%) and the highest grade (4/8) seen at low-level ODE. Although there was a lameness prevalence of 27% in Grand Prix dressage and 23% in 5* TDE it must be borne in mind that this was episodic in the majority, especially in Grand Prix dressage; it was low-grade and generally only observed during movements which were biomechanically demanding, for example half-pass or piaffe, and therefore these horses were potentially judged more stringently than those competing at low-level ODE. It is unclear whether the prevalence of an abnormal canter, 22% in low-level ODE and 15% in 5* TDE, reflects musculoskeletal discomfort, training or a combination thereof. A 5* level Fédération Equestre Internationale (FEI) event judge and Fellow of the British Horse Society commented that in eventing dressage “we see too many horses ridden from hand to leg, rather than from leg to hand … with consequent stiffening of the back … and lack of adjustability of stride; many competitors have to address some basic principles in their methods” [39], suggesting that training is a contributing factor.

### 4.3. Displacement of the Bit

There was a low frequency of occurrence of “bit pulled through to one side” in the non-lame horses compared with lame horses or those with abnormalities of canter. In a study of 150 horses undergoing investigation of low-grade lameness or poor performance “bit pulled through to one side” was observed in 28%, with riders usually indicating that this was associated with asymmetrical rein tension [12]. This was reduced to 3% after diagnostic anaesthesia ± improved saddle fit had substantially reduced musculoskeletal pain. Riders and trainers commonly attribute asymmetric rein tension to the rider, but clearly this can be induced by the horse as an adaptation to musculoskeletal or oral pain. The precise mechanism by which the horse shifts the bit to one side is unknown. It has long been recognised that if a Standardbred racehorse was “on a line”, i.e., there was asymmetrical rein tension, this usually reflected lameness [40], although this may be induced by the driver attempting to keep a horse straight within the shafts. More recently this subjective relationship has been verified objectively [41].

### 4.4. Head behind the Vertical

It was disturbing to observe the high prevalence of the head behind the vertical >10° for at least 10s at all levels in the current study in both the non-lame horses and those with mild lameness. There is additional evidence that in pure dressage at Grand Prix level [42,43,44] and at British Dressage preliminary, novice or elementary levels [45], during either warm-up or during the competition, there is a high prevalence of horses being worked with their heads behind a vertical position. Similar observations have been made during warm-up for 2* to 4* international showjumping competitions [46]. This presumably reflects how the horses are being trained, with a likely lack of awareness of how this posture influences the biomechanics of whole horse movement [47]. The position of the head and neck influences a horse’s ability to engage the muscles of the thoracic sling, the range of motion of the thoracolumbosacral region and the movement patterns of the hindlimbs [48,49]. This undesirable trend may also encourage restrictive and/or coercive riding which may predispose to the development of conflict behaviours and/ or musculoskeletal discomfort.

### 4.5. Mouth Opening and Tail Swishing

In the current study there was a disturbingly high frequency of repeated mouth opening with separation of the teeth and tail swishing especially at Grand Prix dressage and 5*TDE. Several recent studies have highlighted that some of the behavioural signs described in the FEI guidelines [50] as evasions, resistances and disobedience (for example repeated tail swishing and mouth opening) are either going unnoticed by judges, ignored or not penalised [29,30,45]. Opening of the mouth is designated by the FEI as an evasion without ‘active resistance or disobedience’ [51]. The use of a restrictive noseband, for example, a crank cavesson) may reduce the extent of and/or frequency of occurrence of mouth opening and thus may mask a horse’s conflict, discomfort and pain. Judges need to be more aware of possible underlying causes of mouth opening.

Repeated mouth opening may be the result of primary oral discomfort; for example, buccal ulceration secondary to sharp enamel points of the upper molar teeth [52]; periapical tooth root infection [53]; a laceration at a commissure of the mouth [52,54,55,56,57]; discomfort created by the size of the bit(s) relative to the internal dimensions of the oral cavity and the size of the tongue [58], bit shape, rough edges of the canons of the bit, a loose ring snaffle pinching at the junction of the canon of the bit with the ring. Alternatively, it may reflect discomfort created by an excessively tight noseband [59,60]; or discomfort caused by the rider’s rein cues; or secondary to other pain (non-oral). There was no opportunity in the current study to perform an oral examination or to assess the suitability of the bit and bridle. The Grand Prix dressage horses underwent a statutory assessment of noseband tightness, but the method by which this was performed [61] does not prevent there being excessive pressure on the nasal bones or the rami of the mandibles.

### 4.6. Differentiation between Primary Musculoskeletal Pain and Conflict Behaviour

In equitation, the International Society for Equitation Science has defined conflict behaviours as those caused by application of simultaneous opposing signals (conflicting cues such as go and stop/slow) such that the horse is unable to offer any learned responses sufficiently and is forced to endure discomfort from relentless rein and leg pressures [62]. Similarly, conflict behaviour may result from incorrect negative reinforcement, such as the reinforcement of inconsistent responses or lack of removal of pressure.

Conflict behaviours were further described as a set of unwelcomed responses of varying duration that may be characterized by hyper-reactivity and arise largely through confusion [63]. They include but are not limited to, champing the bit, tail-swishing, biting, kicking, rearing and bucking and are thought to have their origins in intraspecific antagonistic behaviours and counter-predator responses [63]. There has been a tendency of those performing observational studies in ridden horses to attribute some aspects of behaviour to conflict behaviour, without consideration of underlying pain [44,45,64,65,66,67]. Indeed, there is considerable cross-over between these behaviours and those that are often observed in ridden horses with musculoskeletal pain [7,10]. Differentiation between pain, rider errors and tack-related problems is not always straightforward [66,68,69,70,71].

In the current study a minority of the non-lame horses showed repeated tail swishing, opening of the mouth with separation of the teeth for at least 10s, a head tilt, an intense stare for 5s or more, or the ears being behind vertical for 5s or more. These may be a manifestation of conflict behaviour.

There are some circumstances when it may be challenging to differentiate between conflict behaviour and pain or a combination. For example, when performing piaffe, a biomechanically demanding movement, a horse may show an irregular hindlimb rhythm, with unequal height steps, put its ears back, repeatedly swish the tail (often in synchrony with spur cues), open the mouth repeatedly (often in rhythm with the steps, therefore possibly being influenced by movement of a rider’s hands [72] ± oral pain induced by the bit), and the head may be persistently behind a vertical position [29]. The question is whether the gait irregularities are because of pain, muscle weakness (reflecting previous training), or lack of strength and coordination, and how much the behaviours are influenced by inadvertent conflicting cues, and a possible influence of a double bridle, or a combination of factors including discomfort.

A poor rider may induce conflict behaviours by the application of conflicting cues however horses vary in their tolerance to either poor riding or pain, and each horse reacts differently [71]. It must be borne in mind that the horses in the current study were ridden by riders with a large range of ability, experience and skill. A horse may be experiencing stress despite showing relatively subtle signs, for example ears back and an intense stare, rather than more obvious signs such as stopping spontaneously. Improvement in a rider’s position and application of cues may improve a horse’s performance, but in the presence of pain behaviours of the RHpE will persist, and veterinary investigation may be merited.

### 4.7. The Relationship between Gait Abnormalities and RHpE Scores

In the current study the median RHpE score was 4/24, but the range 0–12/24 was disturbingly wide, with the high scores predominating at low-level ODE. The high prevalence of continuous lameness, up to grade 4/8, at low-level ODE was concerning. The social licence to operate in equestrian sports is under increasing scrutiny [73,74,75,76], with the focus largely being on the welfare of upper-level competition horses. This study indicates that there is a need to improve standards in low-level competition.

Approximately 80% of lame horses exhibited hindlimb lameness, often bilateral. It has been previously demonstrated that even with training, coaches are poor at recognising hindlimb lameness [77]. Lameness grading in horses with lameness in more than one limb is potentially inaccurate [33]. It is important to recognise features reflecting pain-induced compromises in hindlimb gait such as reduced step length, increased duty factor, reduced range of motion of the thoracolumbosacral region, reduced hindlimb impulsion and engagement, reduced suspension or absence of suspension and changes in speed [34,78,79]. Objective gait analysis relies on analysis of asymmetry, the results of which can be misleading if lameness is bilaterally symmetrical. These observations emphasise the potential value of the RHpE for identifying horses which may be experiencing musculoskeletal discomfort, with the potential to compromise performance.

### 4.8. Limitations

This study had some limitations. The gait was only assessed subjectively, however currently there are no objective methods of evaluating canter or transitions between paces. For both live horse and video assessments it was not possible to evaluate horses directly from behind and from in front on both the left and the right reins, which is the ideal practice for subjective gait analysis. The RHpE was applied by a single observer. However, previous studies have demonstrated good repeatability by a single observer [7,13] and among observers [9,32,80]. Positive results for lameness or the display of a behaviour of the RHpE were only assigned if a result was unequivocal. There was variable rider skill. Tack fit could not be assessed. An ill-fitting saddle [6] and rider force distribution [6,81,82,83] may influence both RHpE scores and gait. It was not possible to assess the mouth for presence of oral lesions. There is the potential for oral lesions to alter head position and stability, mouth opening and position of the bit. Noseband type was recorded but tightness could not be assessed. The size of the bit could not be evaluated accurately. It is not known whether horses would have behaved identically if evaluated in their home environments and the role of stress, if any, related to transport, stabling and the competition environments. However, those horses competing in Grand Prix dressage and 5* TDE would certainly be accustomed to these circumstances.

### 4.9. Future Considerations

Based on the behavioural findings in the non-lame horses in the current study it would be valuable to evaluate ridden horse behaviour using the RHpE in a large group of horses which were objectively determined to have asymmetry of gait outside the ‘normal ranges’ [20,21,84] but which were perceived by their riders to be performing satisfactorily. A low-grade gait abnormality may be seen in competition horses which does not necessarily deteriorate during competition [16] or limit performance [85]. In a recent study of dressage horses, event horses and showjumpers competing in the USA at international level, 89% were lame based on objective evaluation and 88% were lame based on subjective evaluation [85]. No correlation was seen between lameness and performance parameters. However, this contrasts with studies which have shown a negative correlation between RHpE scores and performance in 5*TDEs [28], low-level ODEs [31] and Grand Prix dressage [29,30].

It must also be borne in mind that rideability, the responsiveness of a horse to a rider’s cues, may be influenced by many factors including primary musculoskeletal pain; oral lesions; other systemic pain (e.g., ocular pain, gastrointestinal pain), tack fit for the horse; rider skill, balance, coordination, fitness, position (influenced by saddle fit for the rider), symmetry (and influence of previous injury), correct application of cues (and the influence of trainers and peers); rider morphology and size, and force distribution; use of so-called training aids; and environmental conditions (for example, heat or cold, wind, rain, insects, other horses, noise, other animals, people).

## 5. Conclusions

There was a significant difference in prevalence of most of the 24 behaviours of the RHpE in non-lame compared with lame horses. Abnormal behaviour during ridden exercise should not necessarily be dismissed as conflict behaviour; pain should be considered as a potential underlying cause. Improved recognition of lameness and pain-related behaviour is especially important at low-level competition. Reasons for repeated mouth opening, seen particularly in upper-level competitions, should be investigated. The potential adverse effects of training a horse with the head behind the vertical need to be recognised across the equine industry.

The ultimate aim of riding and training a horse is the development of a harmonious relationship between the horse and rider, with the horse responding to barely perceptible cues, willingly and without excessive tension, moving freely forwards with good quality paces, without undue restriction from a rider, so that the horse progressively develops neuromotor pathways and musculoskeletal strength and coordination appropriate for the tasks it has to perform. This harmonious picture was largely observed in the non-lame horses in the current study. Increased awareness of what a pain-free horse should look like, based on the results of this study, may improve equine welfare and performance, and rider comfort, confidence and safety, and improve training practices.

## Figures and Tables

**Table 1:** Lameness grade (0–8) and Ridden Horse Pain Ethogram (RHpE) scores (0–24) according to event types: Grand Prix dressage tests (n = 211), warm-up for dressage at 5* three-day events (TDE) (n = 137), and dressage phase of low-level one-day events (ODE) (n = 1010). Significant results are highlighted in bold.

	Grand Prix Dressage	5* TDE	Low-Level ODE	Kruskal–Wallis *Χ*^2^; *p*-Value	Dunn’s Test Pairwise *p*-Value
**Lameness grade**				27.3; **<0.001**	Grand Prix—5* TDE: 0.59 Grand Prix—Low-level ODE: **0.002** 5* TDE—Low-level ODE: **<0.001**
Median	0	0	0		
Interquartile range	0, 2	0, 0	0, 1		
Range	0, 3	0, 4	0, 4		
**RHpE score**				15.6; **<0.001**	Grand Prix—5* TDE: 1.00 Grand Prix—Low-level ODE: **0.001** 5* TDE—Low-level ODE: 0.089
Median	3	3	4		
Interquartile range	2, 5	2, 5	2, 6		
Range	0, 9	0, 9	0, 12		

**Table 2:** The frequency of occurrence (number and percentage) for each of the 24 behaviours of the Ridden Horse Pain Ethogram according to event type: Grand Prix dressage (n = 211), warm-up for dressage phase of 5* three-day events (TDE) (n = 137), and dressage phase of low-level one-day events (ODE) (n = 1010). Significant results are highlighted in bold with a Bonferroni adjusted *p*-value < 0.002.

Behaviour	Grand Prix Dressage	5* TDE	Low-Level ODE	*Χ^2^* Test Statistic(2); *p*-Value	Cramér’s V (95% CI)	Effect Size
Head movement up and down, not in synchrony with the trot rhythm	4 (1.9%)	7 (5.1%)	174 (17.2%)	Fisher’s exact *p*-value **<0.001**	0.18 (0.12, 0.23)	weak to moderate
Repeated head tilt	42 (19.9%)	77 (56.2%)	402 (39.8%)	49.7; **<0.001**	0.19 (0.13, 0.24)	weak to moderate
Front of head in front of vertical ≥ 30° ≥ 10s	0 (0.0%)	1 (0.7%)	52 (5.1%)	Fisher’s exact *p*-value **<0.001**	0.10 (0.03, 0.16)	weak
Front of head behind vertical ≥ 10° for ≥ 10s	146 (69.2%)	88 (64.2%)	599 (59.3%)	7.7; 0.021	0.06 (0.00, 0.12)	weak
Head moved from side to side	6 (2.8%)	14 (10.2%)	97 (9.5%)	10.6; 0.005	0.08 (0.00, 0.13)	weak
Ears back behind vertical ≥ 5s	65 (30.8%)	26 (19.0%)	361 (35.7%)	16.0; **<0.001**	0.10 (0.03, 0.15)	weak
Eyes partially closed 2–5s	1 (0.5%)	0 (0.0%)	13 (1.3%)	Fisher’s exact *p*-value 0.423	0.02 (0.00, 0.08)	weak
Repeated exposure of the sclera	16 (7.6%)	31 (22.6%)	62 (6.1%)	44.5; **<0.001**	0.18 (0.12, 0.23)	weak to moderate
Intense stare ≥ 5s	85 (40.3%)	55 (40.1%)	470 (46.5%)	4.16; 0.125	0.04 (0.00, 0.10)	weak
Mouth open with separation of the teeth for ≥ 10s	151 (71.6%)	61 (44.5%)	287 (28.4%)	143.8; **<0.001**	0.32 (0.27, 0.38)	moderate to strong
Repeated exposure of the tongue	19 (9.0%)	11 (8.0%)	79 (7.8%)	0.3; 0.848	0.00 (0.00, 0.04)	weak
Bit pulled through to one side	0 (0.0%)	6 (4.4%)	160 (15.8%)	Fisher’s exact *p*-value **<0.001**	0.19 (0.13, 0.24)	weak to moderate
Crooked tail, or clamped to midline	12 (5.7%)	31 (22.6%)	125 (12.4%)	22.0; **<0.001**	0.12 (0.06, 0.17)	weak
Repeated tail swishing not in synchrony with spur cues	83 (39.3%)	52 (38.0%)	212 (21.0%)	43.2; **<0.001**	0.17 (0.12, 0.23)	weak to moderate
Rushed gait or iregular speed	0 (0.0%)	2 (1.5%)	676 (6.6%)	Fisher’s exact *p*-value **<0.001**	0.12 (0.05; 0.17)	weak
Gait too slow	0 (0.0%)	0 (0.0%)	0 (0.0%)	-	-	-
Crooked, on 3 tracks	3 (1.4%)	6 (4.4%)	287 (28.4%)	101.7; **<0.001**	0.27 (0.22, 0.32)	moderate
Repeated incorrect strike off into canter /disunited	1 (0.5%)	3 (2.2%)	37 (3.7%)	Fisher’s exact *p*-value 0.0273	0.06 (0.00, 0.11)	weak
Spontaneous change of gait	24 (11.4%)	4 (2.9%)	104 (10.3%)	Fisher’s exact *p*-value 0.007	0.07 (0.00, 0.12)	weak
Repeated stumbling or bilateral hindlimb toe drag	29 (13.7%)	0 (0.0%)	378 (37.4%)	Fisher’s exact *p*-value **<0.001**	0.28 (0.23, 0.34)	moderate
Spontaneous change of direction; spooking	4 (1.9%)	2 (1.5%)	29 (2.9%)	Fisher’s exact *p*-value 0.651	0.00 (0.00, 0.07)	weak
Reluctance to go forwards	5 (2.4%)	2 (1.5%)	40 (4.0%)	Fisher’s exact *p*-value 0.244	0.03 (0.00, 0.09)	weak
Rearing	5 (2.4%)	1 (0.7%)	6 (0.6%)	Fisher’s exact *p*-value 0.048	0.06 (0.00, 0.11)	weak
Bucking	3 (1.4%)	4 (2.9%)	20 (2.0%)	Fisher’s exact *p*-value 0.620	0.00 (0.00, 0.06)	weak

*Χ*^2^ test statistic (2) = Chi-squared test statistic with 2 degrees of freedom; CI = confidence interval. Differences in effect size categories (weak, weak-moderate, moderate and moderate to strong) are highlighted by variable shading.

**Table 3:** The frequency of occurrence of each of the 24 behaviours of the Ridden Horse Pain Ethogram in lame horses (n = 553), non-lame but with an abnormal canter (n = 255), and horses with no lameness and a normal canter (n = 550). Significant results are highlighted in bold with a Bonferroni adjusted *p*-value < 0.002.

Behaviour	Abnormal Canter	Lame	Non-Lame	*Χ*^2^ Test Statistic(2); *p*-Value	Cramér’s V (95% CI)	Effect Size
Head movement up and down, not in synchrony with the trot rhythm	38 (14.9%)	104 (18.8%)	43 (7.8%)	28.7; **<0.001**	0.14 (0.08, 0.19)	weak
Repeated head tilt	105 (41.2%)	231 (41.8%)	185 (33.6%)	8.8; 0.013	0.07 (0.00, 0.12)	weak
Front of head in front of vertical ≥ 30° ≥ 10s	3 (1.2%)	40 (7.2%)	10 (1.8%)	27.8; **0.001**	0.14 (0.08, 0.19)	weak
Front of head behind vertical ≥ 10° for ≥ 10s	165 (64.7%)	341 (61.7%)	327 (59.5%)	2.07; 0.356	0.007 (0.00, 0.08)	weak
Head moved from side to side repeatedly	27 (10.6%)	69 (12,5%)	21 (3.8%)	27.8; **<0.001**	0.14 (0.08, 0.19)	weak
Ears back behind vertical ≥ 5s	76 (29.8%)	272 (49.2%)	104 (18.9%)	115.6; **<0.001**	0.29 (0.23, 0.34)	moderate
Eyes partially closed 2–5s	1 (0.4%)	12 (2.2%)	1 (0.2%)	11.9; 0.003	0.09 (0.00, 0.14)	weak
Repeated exposure of the sclera	20 (7.8%)	49 (8.9%)	40 (7.3%)	0.96; 0.620	0.00 (0.00, 0.06)	weak
Intense stare ≥ 5s	112 (43.9%)	343 (62.0)	155 (28.2%)	127.8; **<0.001**	0.30 (0.25, 0.36)	moderate to strong
Mouth open with separation of the teeth for ≥ 10s	81 (31.8%)	225 (40.7%)	193 (35.1%)	7.1; 0.029	0.06 (0.00, 0.12)	weak
Repeated exposure of the tongue	22 (8.6%)	51 (9.2%)	36 (6.5%)	2.8; 0.243	0.02 (0.00, 0.08)	weak
Bit pulled through to one side	31 (12.2%)	100 (18.1%)	35 (6.4%)	35.3; **<0.001**	0.16 (0.10, 0.21)	weak to moderate
Tail crooked, or clamped to midline	32 (12.5%)	82 (14.8%)	54 (9.8%)	6.4; 0.041	0.06 (0.00, 0.11)	weak
Repeated tail swishing not in synchrony with spur cues	68 (26.7%)	170 (30.7%)	109 (19.8%)	17.5; **<0.001**	0.11 (0.04, 0.16)	weak
Rushed gait or irregular speed	16 (6.3%)	35 (6.3%)	18 (3.3%)	6.3; 0.044	0.06 (0.00, 0.11)	weak
Gait too slow	0 (0.0%)	0 (0.0%)	0 (0.0%)	-	-	-
Crooked, on 3 tracks	62 (24.3%)	169 (30.6%)	65 (11.8%)	58.0; **<0.001**	0.20 (0.15, 0.26)	weak to moderate
Repeated incorrect strike off in canter/disunited	15 (5.9%)	20 (3.6%)	6 (1.1%)	14.8; **0.001**	0.10 (0.03, 0.15)	weak
Spontaneous change of gait	23 (9.0%)	58 (10.5%)	51 (9.3%)	0.6; 0.726	0.00 (0.00, 0.05)	weak
Repeated stumbling or bilateral hindlimb toe drag	59 (23.1%)	311 (56.2%)	37 (6.7%)	329.1; **<0.001**	0.49 (0.44, 0.54)	strong
Spontaneous change of direction; spooking	9 (3.5%)	15 (2.7%)	11 (2.0%)	1.69; 0.429	0.00 (0.00, 0.07)	weak
Reluctance to go forwards	11 (4.3%)	27 (4.9%)	9 (1.6%)	9.4; 0.009	0.07 (0.00, 0.13)	weak
Rearing	3 (1.2%)	5 (0.9%)	4 (0.7%)	Fisher’s exact *p*-value 0.808	0.00 (0.00, 0.04)	weak
Bucking	7 (2.7%)	15 (2.7%)	5 (0.9%)	Fisher’s exact *p*-value 0.044	0.05 (0.00, 0.11)	weak

*Χ*^2^ test statistic (2) = Chi-squared test statistic with 2 degrees of freedom; CI = confidence interval. Differences in effect size categories (weak, weak-moderate, moderate, moderate to strong and strong) are highlighted by variable shading.

**Table 4:** Summary of the frequency of occurrence of the 24 behaviours of the Ridden Horse Checklist for horses without lameness, n = 805, for horses competing in Grand Prix Dressage, 5* three-day events, and low-level one-day events.

Behaviour	Frequency	
	Number	%
Stable head position, not repeatedly moved up and down	724	89.9
Absence of repeated head tilt	515	64.0
Head not in front of vertical (>30°) for ≥ 10s	792	98.4
Head not behind vertical (>10°) for ≥ 10s	313	38.9
Stable head position, not moved repeatedly from side to side	757	94.0
Ears forward or erect or rotated to side, but not behind vertical for ≥ 5s	625	77.6
Eye lids not closed or half closed for 2–5s	803	99.8
Sclera not exposed repeatedly	745	92.5
“Engaged” expression; absence of an intense stare (also described as a glazed expression or ‘zoned out’) for ≥5s	538	66.8
Mouth shut (teeth apposed) most of the time; not opening with separation of teeth, for ≥ 10s	531	66.0
Tongue not observed more than once outside oral cavity	747	92.8
Bit positioned symmetrically in horse’s mouth	739	91.8
Tail not clamped tightly to middle or persistently held to one side	719	89.3
Absence of repeated tail swishing	628	78.0
Rhythmical gaits with constant speed; frequency of trot steps ≥35 <40 steps/15s	771	95.8
Gait not too slow (frequency of trot steps ≥35/15s)	805	100.0
Hindlimbs follow tracks of forelimbs in all gaits	678	84.2
Canter: not more than one incorrect strike off with wrong forelimb leading; absence of change of leg in front and/or behind (not disunited or cross-cantering)	784	97.4
No spontaneous changes of gait	731	90.8
Not more than one forelimb or hindlimb stumble; absence of repeated bilateral hindlimb toe drag	709	88.1
Follows rider’s directional cues; no sudden change of direction against rider’s cues; or spooking	785	97.5
Responds willingly to cues with the absence of repeated strong leg cues or verbal encouragement; no stopping spontaneously	785	97.5
No rearing	798	99.1
No bucking	793	98.5

## Data Availability

Anonymised data are available from the authors upon reasonable request.
